# Nutritional Approach in Selected Inherited Metabolic Cardiac Disorders—A Concise Summary of Available Scientific Evidence

**DOI:** 10.3390/nu15224795

**Published:** 2023-11-16

**Authors:** Alina Costina Luca, Ioana-Alexandra Pădureț, Viorel Țarcă, Simona Georgiana David, Dana Elena Mîndru, Solange Tamara Roșu, Eduard Vasile Roșu, Heidrun Adumitrăchioaiei, Jana Bernic, Elena Cojocaru, Elena Țarcă

**Affiliations:** 1 Pediatrics Department, “Grigore T. Popa” University of Medicine and Pharmacy, 700115 Iasi, Romania; alina.luca@umfiasi.ro (A.C.L.); paduret.alexandra@gmail.com (I.-A.P.); mindru.dana@umfiasi.ro (D.E.M.); eduard.rosu@umfiasi.ro (E.V.R.); 2 Saint Mary Emergency Hospital for Children, 700309 Iasi, Romania; simona.david23@gmail.com (S.G.D.); ad.heidi91@gmail.com (H.A.); 3 Department of Preventive Medicine and Interdisciplinarity, “Grigore T. Popa” University of Medicine and Pharmacy, 700115 Iasi, Romania; 4 Nursing Department, “Grigore T. Popa” University of Medicine and Pharmacy, 700115 Iasi, Romania; solange.rosu@umfiasi.ro; 5 Discipline of Pediatric Surgery, “Nicolae Testemițanu” State University of Medicine and Pharmacy, 2025 Chisinau, Moldova; jana.bernic@usmf.md; 6 Department of Morphofunctional Sciences I—Pathology, “Grigore T. Popa” University of Medicine and Pharmacy, 700115 Iasi, Romania; 7 Surgery II Department—Pediatric Surgery, “Grigore T. Popa” University of Medicine and Pharmacy, 700115 Iasi, Romania; tarca.elena@umfiasi.ro

**Keywords:** inherited metabolic cardiac disorders, diet, supplements

## Abstract

Inborn errors of metabolism (IMDs) are a group of inherited diseases that manifest themselves through a myriad of signs and symptoms, including structural or functional cardiovascular damage. The therapy of these diseases is currently based on enzyme-replacement therapy, chaperone therapy or the administration of supplements and the establishment of personalized dietary plans. Starting from the major signs identified by the pediatric cardiologist that can indicate the presence of such a metabolic disease—cardiomyopathies, conduction disorders or valvular dysplasias—we tried to paint the portrait of dietary interventions that can improve the course of patients with mitochondrial diseases or lysosomal abnormalities. The choice of the two categories of inborn errors of metabolism is not accidental and reflects the experience and concern of the authors regarding the management of patients with such diagnoses. A ketogenic diet offers promising results in selected cases, although, to date, studies have failed to bring enough evidence to support generalized recommendations. Other diets have been successfully utilized in patients with IMDs, but their specific effect on the cardiac phenotype and function is not yet fully understood. Significant prospective studies are necessary in order to understand and establish which diet best suits every patient depending on the inherited metabolic disorder. The most suitable imagistic monitoring method for the impact of different diets on the cardiovascular system is still under debate, with no protocols yet available. Echocardiography is readily available in most hospital settings and brings important information regarding the impact of diets on the left ventricular parameters. Cardiac MRI (magnetic resonance imaging) could better characterize the cardiac tissue and bring forth both functional and structural information.

## 1. Introduction

**Figure 1:**
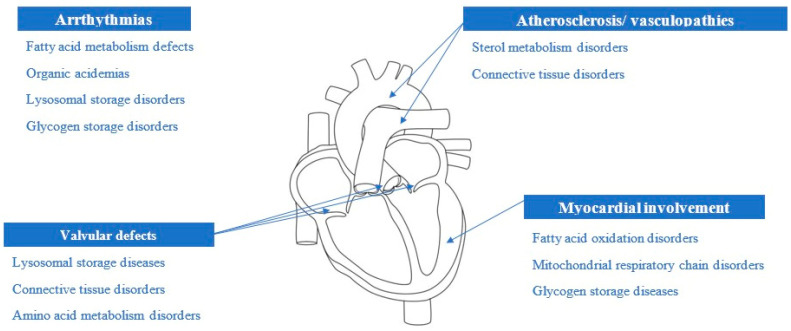
Etiological suspicions depending on the cardiac involvement (original picture).

Cardiovascular involvement is a known feature of a myriad of inherited metabolic disorders (IMDs). Cardiac manifestations may occur in the absence of any other signs or symptoms, or they may be part of a multisystem disorder. In order to suspect an impairment of metabolism in a patient with cardiovascular disease, it is useful to first broadly summarize the relationship between the type of cardiac injury and the nature of the metabolic error (Figure 1).

A broad classification of IMDs comprises three groups: (1) substrate accumulation, in which an intermediary compound of metabolism is accumulated, leading to either acute or progressive intoxication, and such is the case in organic acidurias; (2) energy metabolism disorders, which affect organs that require high energy expenditure for proper functioning, such as the heart, liver, brain and muscles, and are caused by mitochondrial disorders; and (3) complex molecules diseases, which are disorders of synthesis or catabolism, like lysosomal storage disorders or peroxisomal diseases.

Although a well-defined protocol for diagnosing a cardiovascular disease in the setting of a metabolic disorder is yet to be established, there are a few tell-tale signs that should give rise to suspicion. The presence of hypoglycemia in a patient with cardiomyopathy may well be caused by a fatty acid metabolism defect, while the couple cardiomyopathy–hypotonia is highly indicative of a systemic muscle disease in the setting of a mitochondrial disease or a disorder of glycosylation. Dysmorphic features, cardiomyopathy, skeletal abnormalities and hepatosplenomegaly usually occur together in mucopolysaccharidoses. Cardiac conduction abnormalities pose a tricky question since they can be found in both storage disorders and primary mitochondrial diseases [1].

The purpose of this article is not to present the diagnostic criteria for each of the diseases found in the categories mentioned above, but to offer a broad perspective over each one of them and insight into the dietary interventions required once the proper diagnosis has been made. Table 1 summarizes the inherited metabolic disorders affecting the cardiovascular system and proposed dietary interventions [2,3,4].

## 2. Materials and Methods

We conducted a literature review by using PubMed and ScienceDirect to retrieve articles referring to the supplements and diets used to manage patients with inherited mitochondrial cardiovascular diseases and lysosomal storage diseases. We did our research in a staged manner, firstly selecting articles about inherited metabolic diseases with a cardiac phenotype or functional modifications, and afterwards we selected articles on dietary interventions that proved to alleviate cardiac burden. The primary objective of our paper is to offer the pediatric cardiologist an overview of nutritional approaches in inherited metabolic cardiac diseases.

## 3. Overview of Cellular Energy Generation

Glucose is readily accessible to cells through facilitated diffusion, aided by various isotypes of glucose transporters found in different cell types. The concentration of glucose in the bloodstream increases following a meal or as a result of hepatic gluconeogenesis. This process involves the liver releasing glucose during periods of fasting, which enables tissues to absorb glucose and convert it into pyruvate through glycolysis. Following this, mitochondria have the ability to consume glucose in the form of pyruvate to generate adenosine triphosphate (ATP). In physiological circumstances, the transportation of pyruvate across the double mitochondrial membrane occurs through the use of the mitochondrial pyruvate carrier (MPc). The process of oxidative phosphorylation is attenuated by a lack of oxygen, resulting in a reduction in the net energy production. In the absence of oxygen, energy is generated through anaerobic glycolysis, leading to the production of lactate as the final product. In hepatocytes, lactate has the ability to undergo conversion into pyruvate and glucose through an alternative form of lactate dehydrogenase when oxygen levels are plentiful. This process, however, leads to a net decrease in ATP production. Alternatively, when the activity of MPc proteins, which impede the transportation of pyruvate inside the mitochondria, is suppressed, the mitochondria undergo metabolic reprogramming and rely predominantly on fatty acids and glutamine as energy sources. The glutaminolysis pathway facilitates the oxidation of glutamine within the mitochondria, hence promoting the activation of the Krebs cycle. This process leads to the production of a-ketoglutarate or pyruvate through the involvement of malic enzymes [14].

Interestingly, it has been observed that cells experience a decline in their capacity to effectively utilize surplus pyruvate when exposed to elevated levels of glucose. This phenomenon ultimately results in cellular glucotoxicity, which is brought about by the activation of many pathways, including the polyol pathway, as well as protein kinase c, the increased production of advanced glycation end products and alterations in the hexosamine pathway flux. Subsequently, despite the proposition that an abundance of glucose promotes increased energy generation, it is noteworthy that the mitochondria are susceptible to the harmful byproducts generated by glucotoxicity, leading to mitophagy and subsequent cellular demise. This suggests that maintaining glucose homeostasis is essential for the optimal functioning of mitochondria and cells [14].

Fatty acids (FAs) serve as the primary metabolic substrates for the mitochondria of cardiac and skeletal muscles, meeting their energy requirements. Fatty acids (FAs) are produced from white adipose tissue either as albumin-bound FAs or by the breakdown of very-low-density lipoprotein (VLDL) mediated by lipoprotein lipase. Following this, fatty acids (FAs) are transported into the mitochondria or peroxisomes, where they undergo catabolism through β- and α-oxidation processes. The rate of β-oxidation and the concentrations of its primary product, acetyl-CoA, determine the energy requirements of cells. When there is insufficient ATP to meet the heightened cellular demands, it leads to an increase in the activity of the tricarboxylic acid (TCA) cycle and oxidative phosphorylation (OXPHOS). In a similar fashion, it has been observed that the decrease in NAdH and acetyl-CoA levels results in the activation of a β-oxidation flux. In contrast, the activity of cPT1 plays a crucial role in determining the rate of β-oxidation in cardiac or skeletal muscle. As previously indicated, the products resulting from β-oxidation include acetyl-CoA, which subsequently enters the tricarboxylic acid (TCA) cycle, as well as NADH and FADH2. These electron carriers are essential for maintaining the electron flow in the electron transport chain (ETC), so establishing the requisite gradient for F-ATPase is necessary to generate ATP, the currency of cellular energy [14].

## 4. Nutritional Approaches in Primary Mitochondrial Diseases

Primary mitochondrial diseases (PMDs) are a heterogeneous group of afflictions caused by anomalies in the mitochondrial respiratory chain or in cellular oxidative phosphorylation [15,16,17]. Mitochondrial DNA (mtDNA) codes for part of the subunits forming the five complexes, and the remaining parts are synthesized from nuclear gene transcripts [18,19,20]. The diverse manifestations of PMDs in tissues reside in heteroplasmy—the proportion of normal and mutated mtDNA within cells—which allows for disease manifestations to occur when the mutated DNA overcomes the normal DNA, leading to considerable variability and preferential lesions [5,6,21,22,23]. Needless to say, cardiac cells, which cannot further divide, are subject to higher degrees of mutated mtDNA accumulation. An overview concerning PMDs with cardiovascular involvement is summarized in Table 2.

Dietary requirements for individuals with mitochondrial diseases can vary depending on the specific type of mitochondrial disease, severity, age, growth and other individual factors [18,19,42]. Currently, there is no proof in favor of a medication-based treatment for mitochondrial illness. This is due, in part, to the dearth of randomized controlled clinical trials (RCT) examining PMD treatments and the complexity of the illness. Dietary supplements (DS) are a common therapeutic treatment alternative that has emerged as a crucial standard of care [43]. Despite being perhaps the most widely used intervention, there are few RCTs that support the effectiveness and safety of dietary supplements as a treatment option in the literature, making it challenging for doctors to make recommendations that are supported by the available data. The Mitochondrial Medicine Society does, in fact, only propose a small number of supplement kinds, depending on the type of condition and based on consensus rather than clinical outcomes [13].

Different types of diets have been studied as possible nonpharmacological therapies aimed at improving mitochondrial function, with an emphasis on the ketogenic diet [44,45,46].

The ketogenic diet, often known as the KD, is an eating plan that is low in carbohydrates and rich in fat. It encourages ketogenesis, which is the production of ketone bodies from fat storage, as well as betaoxidation, which is the breakdown of fat. The amounts of fat and protein that are included in various low-carbohydrate diets, not to mention their overall calorie counts, might vary greatly from one another. Less than a third of the calories in a standard KD come from protein (15–35%), and less than ten percent come from carbohydrates (5–10%). The majority of the calories in a traditional KD come from fat (55–90%). The conventional ketogenic diet recommends consuming each meal in accordance with a predetermined ratio of fat, carbohydrates and protein (such as 4:1 or 3:1) [47,48]. Carbohydrate consumption is restricted to between 10 and 20 g per day on the modified Atkins diet (MAD), in contrast to the classic Atkins diet, which also places restrictions on fat and protein intake. Patients who have behavioral issues and children who are being treated with diets for epilepsy have a higher rate of compliance when the MAD is used rather than the KD [49]. This is because the MAD is more enticing and less restrictive than the KD. In general, KDs are well tolerated and safe, and research has shown that they are effective in the treatment of intractable epilepsy [20,38,50].

Additionally, there have been reports of KD patients seeing beneficial impacts on their cardiac phenotypes [51,52]. A patient who was diagnosed with Leigh encephalopathy and cardiomyopathy when they were 3 months old and began a ketogenic diet when they turned 8 months old was successfully managed, according to the findings presented by Kucharska DW et al. The patient’s clinical, biochemical and echocardiographic state all demonstrated significant improvement during the course of the patient’s long-term follow-up. The authors went as far as to add that no such benefits were noticed after putting the patient on a mitochondrial cocktail [38]. A mitochondrial cocktail is a collection of supplements that are often used in the treatment of mitochondrial illnesses, such as thiamine, coenzyme Q10, arginine, glutathione and alpha-lipoic acid.

There are reports indicating that patients with MELAS syndrome benefited from the KD diet in a manner analogous to that described above [45]. Notably, Fang He et al. [40] disclose the example of a patient who is now 20 years old but was diagnosed with mitochondrial encephalopathy when he was only 9 years old. Before the KD was started, none of the other therapeutic techniques that were tried helped avoid seizures and a general deterioration in the patient’s condition. The authors describe a step-down method in the ketogenic diet, which first started with a 4:1 ratio and subsequently lowered to a 2:1 ratio nine months into the treatment. With the exception of a relapse brought on by the patient’s temporary noncompliance, a significant improvement in the patient’s general state was seen, including an increase in their exercise tolerance.

KDs have also been utilized in some individuals who suffer from both MD and epilepsy, and the short- and long-term follow-up of these patients has revealed encouraging results [16,53]. It is hypothesized that KDs exert their therapeutic effect by promoting mitochondrial biogenesis, boosting mitochondrial function and lowering oxidative stress. Studies have indicated that the KD can lower the frequency and severity of seizures in patients with MD. However, there is a possibility that it also has other benefits [53,54].

Studies have shown that the heart and brain tissue are more responsive to the fuel provided by ketone bodies than they are to glucose, even when both are readily available [2,55,56]. KDs, as a whole, constitute a novel strategy for treating PMD, which justifies the need for additional investigations. Those who are attempting the KD often struggle with adhering to the prescribed diet, in part because of the restrictive nature of the diet, and also because of the adverse effects such as constipation or diarrhea, headaches and muscle cramps. There have also been reports of deleterious effects on the cardiovascular system, such as bradycardia and an increased likelihood of developing atherosclerotic disease [57,58].

## 5. Nutritional Approaches in Lysosomal Storage Diseases

A group of several inherited diseases known as lysosomal storage diseases (LSDs) are caused by specific defects in lysosomal enzymes. The nature of the accumulated substances is used to classify the disorders. Lysosomes act as the cell’s digestive system, breaking down material that is taken in from outside the cell as well as cellular waste. Each and every enzyme present in the lysosome is an acid hydrolase that operates optimally at a specific acidic pH of approximately 5, which is specific to lysosomes and not the neutral pH of the cytoplasm, which is around 7.2. This acidic pH is exclusive to lysosomes and enables lysosomal hydrolases to have two-fold protection against the uncontrolled breakdown of cytosolic contents. The acid hydrolases generated in the event of lysosomal membrane disintegration would be ineffective in the cytosol’s neutral pH [59]. Variations in the genetic code that produce these lysosomal proteins are the root cause of over 30 distinct hereditary conditions in humans, collectively known as lysosomal storage diseases. These diseases are characterized by the buildup of undigested substances in the lysosomes of affected individuals. The majority of these diseases are due to the insufficient production of individual lysosomal proteins, resulting in a widespread inability of lysosomal enzymes to integrate properly into lysosomes [59]. Amongst the most serious threats for patients with LSDs are respiratory infections, which, considering that the immune system in these patients is impaired, usually have a poor prognosis [59,60]. Alleviating LSDs’ manifestations and ensuring adequate nutrient intake through an appropriate diet is paramount.

There are more than 50 types of lysosomal storage diseases, but the types that occur most often in infants, children and adolescents include Gaucher disease, Niemann–Pick disease, Fabry disease, Tay–Sachs disease, Pompe disease and mucopolysaccharidoses (MPS) diseases (Table 3).

Gaucher disease is a hereditary disease with an autosomal recessive pattern of inheritance caused by mutations in the GBA1 gene, which encodes acid glucosidase (glucocerebrosidase). The disease is characterized by the inadequate activity of the enzyme, leading to the accumulation of glycosphingolipids, especially glucosylceramide, in the lysosomes of various tissues and cells. This accumulation leads to various manifestations of bone and visceral pathologies, and in some, neuronal involvement may occur. Data regarding specific diets for GD are lacking; however, vitamin D and calcium supplementation are mandatory, and low-carbohydrate/high-protein diets seem reasonable considering the disease mechanism. The resting energy expenditure (REE) is increased in patients before receiving ERT, which makes them prone to malnutrition. After enzyme substitution initiation, the REE reaches normal levels, a fact that exposes patients to being overweight if dietary plans are not modified accordingly [67].

Pompe disease, also referred to as GSDII or glycogen storage disease type II (GSDII), is a rare neuromuscular disorder that is inherited in an autosomal recessive manner and can affect individuals of any age. The patients lack the enzyme acid alpha-glucosidase (GAA), responsible for the complete degradation of glycogen to glucose within the lysosome [68,69]. The accumulation of glycogen in the body can lead to significant changes in cell structure, which in turn can cause cell damage and eventually lead to cell death. The severity of this disease is influenced by several factors, including the type of mutations, residual enzyme activity and the age at which symptoms first manifest. Glycogen accumulation in lysosomes is a primary cause of cellular dysfunction, particularly in motor neurons, smooth and skeletal muscle cells and cardiac cells. These changes can eventually impair the function of the affected cells and lead to a range of symptoms and complications [68,69].

In GSDII, the ketogenic diet is proposed as a therapeutic nutritional intervention, aimed at reducing substrates and inducing nutritional ketosis. An alternative to the troublesome KD is the low-carbohydrate high-protein diet, which may improve quality of life [64].

Niemann–Pick disease, a rare neurodegenerative metabolic disease, belongs to the class of lysosomal storage diseases (LSDs). It is a form of hereditary lipidosis that is inherited in an autosomal recessive manner and leads to the buildup of sphingolipids in the body’s cells, particularly reticuloendothelial cells. Six types of Niemann–Pick disease have been reported: A—acute neuropathic, B—visceral, C—chronic neuropathic, D—Nova Scotia variant, E—adult type and F—sea-blue histiocytic disease. Niemann–Pick type C disease is the most widely studied category among all the others.

The initial idea for treating Niemann–Pick type C disease was to try and decrease the amount of cellular cholesterol since this condition leads to an accumulation of cholesterol in lysosomes. Hence, a diet low in cholesterol and drugs used to treat high cholesterol were successful in reducing the overall blood cholesterol and liver cholesterol levels, but there was no improvement in neurological symptoms [66,70]. However, Holler A et al. reported using the KD as a means to obtain seizure control in a patient diagnosed with Niemann–Pick type C disease [71]. In their case, the 17-year-old female patient was treated with miglustat and the KD and was followed-up on for 3 years. Despite not conferring neuroprotection, the KD helped decrease the number of hospitalizations caused by seizures.

Fabry disease (FD) is a rare and progressive lysosomal storage disorder caused by mutations in the GLA gene, which is located on the X chromosome. This genetic mutation leads to the reduced or absent activity of the α-galactosidase A (α-Gal A/GLA) enzyme, resulting in the accumulation of glycolipids, specifically globotriaosylceramide (GL-3, Gb3) and globotriaosylsphingosine (lyso-GL-3), in various tissues and cells. This accumulation causes progressive damage to the organs affected, as well as life-threatening complications and an elevated risk of premature death [59,72,73]. While there is not enough compelling evidence to support a specific dietary plan for FD patients, a low-fermentable oligosaccharide, disaccharide, monosaccharide and polyol (FODMAP) diet has been shown to alleviate gastrointestinal symptoms. To date, there are no available data on the impact of such a diet on FD cardiomyopathy.

## 6. Impact of Nutritional Intervention on Cardiac Function in Patients with Inherited Metabolic Disorders

As stated in the above sections, various diets have been proposed as secondary or primary therapeutic interventions for various inherited metabolic disorders affecting the cardiovascular system. It is therefore appropriate to discuss and summarize the concrete evidence of the impact of said diets on cardiac function.

The ketogenic diet has reportedly been used in a patient with lysosomal storage disease, with significant results in terms of decreasing ventricular hypertrophy and increasing the ejection fraction [4]. However, in this case, which was described by Villamizar-Schiller et al., the ketogenic diet and miglustat were used in combination from the very beginning of the patient’s treatment, and the latter has well-documented effects of reducing ventricular hypertrophy and improving cardiac function by itself. As a result, despite the results that the authors reported, it is difficult to establish a causality link between the KD and ventricular function and structure improvement.

Nevertheless, in their work, Marusic et al. used the KD as the sole available treatment for a patient with Cori disease (a glycogen storage disease) and documented the cardiac effects over the course of 4 years. A significant reduction in cardiac markers, namely pro-BNP and Ck-MB, was noted 12 months after the initiation of the KD. The left ventricular parameters also showed a tendency towards normalization, with a decrease in the left ventricular mass index and wall thickness [5,6].

Comparing the work of the two authors, significant questions arise. The first question concerns the effectiveness of the KD in managing cardiac function in patients with inherited metabolic cardiac disorders in the absence of any other available treatment (such as chaperone therapy or ERT). The second one addresses the issue of proper cardiac monitoring—namely, is a 2D echocardiography evaluation sufficient to characterize the left ventricular parameters and cardiac function, or should a cardiac MRI (CMR) be used instead?

Regarding the effectiveness of the ketogenic diet as the only method of treatment, extrapolating from the case reports in the literature, a beneficial profile emerges, with significant results demonstrating that the KD improves cardiac function in the absence of another specific therapy, both in mitochondrial diseases and in conditions characterized by substrate accumulation.

However, Zweers’ meta-analysis, which summarizes the effects of the KD in mitochondrial diseases, concludes that there are not enough data at this time to issue generalized recommendations for implementing the ketogenic diet in patients with mitochondrial diseases [52].

The paucity of meta-analyses regarding the effectiveness of various diets in managing cardiac function in the setting of an inherited metabolic disease in the pediatric population is an issue that should be promptly addressed.

Regarding the cardiac imagistic method of choice for monitoring patients, there is still no consensus or standardized protocol that establishes the superiority of cardiac MRI over echocardiography or vice versa. If we take into account the fact that, in general, tissue characterization is usually performed through a CMR, we could consider the former as the most indicated method of imaging evaluation. Nevertheless, valvular anatomy and function are better evaluated during echocardiography. A multimodal imaging approach could therefore complete the picture of the cardiac evolution of patients with IMD treated with the various diets mentioned previously.

In this sense, studies that comparatively analyze the evolution of cardiac parameters measured echocardiographically and by MRI could be useful. One must take into account, however, the logistical and socioeconomic restrictions that can make access to a cardiac MRI difficult in certain contexts [74].

Regarding the other diets mentioned in Table 3, corresponding to each lysosomal storage pathology separately, the studies focused on their impact on the quality of life, the improvement in the exercise capacity and the muscle strength of participants. Although the description of the echocardiographic and/or CMR parameters is missing, the improvement in exercise capacity is indirect proof of the normalization of cardiac function. However, prospective studies are needed to evaluate pediatric patients under specific diets from all aspects—quality of life, effort capacity, cognitive function, digestive function, cardiac function, etc. (Table 4).

## 7. Conclusions

As mentioned previously, recent studies in the nutritional area have focused on identifying supplements and diets that can improve the quality of life and prognosis of patients with lysosomal storage diseases or mitochondrial diseases. A consensus does not exist at this moment, the indications being based on studies carried out on small groups of patients, on case reports or on the extrapolation of data on patients with pathologies with a similar physiopathological mechanism. The ketogenic diet has a favorable effect on patients with mitochondrial diseases and those with neurological damage. In lysosomal diseases, the dietary indications take into account the pathophysiological mechanism of the disease itself, recommending the avoidance of foods that can potentiate substrate accumulation or those that can interfere with the metabolism of enzyme-replacement-type medication. The topic of nutrition in inborn diseases of metabolism is still subject to research, taking into account the rarity of cases and the fact that the factor of patient compliance to nutritional interventions is difficult to control. In this article, we tried to provide a concise overview of the data available in the literature. Additional investigations are needed that include the experiences of different populations affected by these diseases, which take into account both socioeconomic and cultural aspects. We strongly recommend that any management plan for patients with inherited metabolic disorders involve genetic counseling; psychotherapy; financial aid from available organizations (in order to secure treatment compliance); and a detailed, mutually agreed follow-up plan that both the doctor and patient find satisfactory. All of the above-mentioned aspects are necessary to ensure long-term monitoring and patient compliance.

## Figures and Tables

**Table 1:** Summary of inherited metabolic disorders associated with cardiovascular anomalies [2,3,4,5,6,7,8,9,10].

Disease Category	Cardiac Manifestations	Red Flags	Dietary Intervention [11,12,13]
Glycogen storage disorders	Hypertrophic cardiomyopathy Conduction abnormalities	Hepatomegaly Hypoglycemia	High-protein diet Modified Atkins diet [7]
Fatty acid oxidation disorders	Hypertrophic/dilated cardiomyopathy	Hypoketotic hypoglycemia; Episodic rhabdomyolysis	Avoidance of fasting Carnitine supplementation Fat-restricted diet and DHA supplementation for long-chain FAMD
Primary mitochondrial diseases	Hypertrophic/dilated cardiomyopathy Arrhythmias Conduction abnormalities	Skeletal muscle symptoms Encephalopathy Seizures Episodic vomiting/ketoacidosis	Dietary supplements Ketogenic diet
Organic acidemias [9]	Cardiomyopathy Arrhythmias	Metabolic decompensation Hearing impairment Renal failure	Avoid catabolism Carnitine supplementation
Lysosomal storage disorders	Valvular abnormalities Conduction abnormalities Large vessel anomalies	Skeletal abnormalities Dysmorphic features Hepatosplenomegaly Corneal clouding Hearing impairment	Vitamin supplementation Ketogenic diet High-protein diet

**Table 2:** Supplement use in cardiovascular mitochondrial diseases [11,24,25,26,27].

Disease Name [28]	Red Flags [29]	Cardiovascular Manifestations [30,31]	Dietary Intervention [21,22,32,33,34,35]
Chronic progressive external ophthalmoplegia	Ptosis Ophthalmoparesis Proximal myopathy	Prolonged intraventricular conduction time Bundle branch blocks Complete AV block	Alpha-lipoic acid Carnitine Coenzyme Q10 Creatine
Kearns–Sayre syndrome (KSS)	Ataxia Hearing loss Myopathy Pigmentary retinopathy Elevated CSF proteins	Atrioventricular block which requires pacemaker implantation	Carnitine Coenzyme Q10 Creatine Folinic acid
Leber’s hereditary optic neuropathy (LHON) [36]	Progressive bilateral visual failure	Hypertrophic cardiomyopathy Atrioventricular conduction abnormalities (long QT, WPW) Left ventricle hypertrabeculation	Coenzyme Q10
Leigh syndrome [37]	Encephalopathy Seizures Hypotonia/ataxia Oculomotor dysfunction Respiratory dysfunction	Hypertrophic cardiomyopathy Conduction abnormalities	Carnitine Coenzyme Q10 Ketogenic diet [12,38]
Mitochondrial myopathy, encephalopathy, lactic acidosis and stroke-like episodes (MELAS) [39,40,41]	Stroke-like symptoms Lactic acidemia Myopathy	Hypertrophic cardiomyopathy Dilated cardiomyopathy Conduction abnormalities	Arginine Carnitine Citrulline Coenzyme Q10 Creatine Niacin
Mitochondrial neurogastrointestinal encephalopathy (MNGIE)	Gastrointestinal dysmotility Polyneuropathy Leukodystrophy	Conduction abnormalities Sudden cardiac death	Coenzyme Q10
Myoclonic epilepsy with ragged-red fibers (MERRF)	Ataxia Myoclonus Generalized seizures	Dilated cardiomyopathy Hypertrophic cardiomyopathy Conduction abnormalities	Coenzyme Q10 Creatine

**Table 3:** Lysosomal storage diseases—cardiac and systemic signs and symptoms. Nutritional approach [23,54,61,62].

Disease Name	Red Flags	Cardiac Manifestations [5]	Dietary Intervention [6]
Gaucher	Hepatosplenomegaly Peripheral blood cytopenia Bone lesions Different degrees of neurologic impairment	Pulmonary hypertension Aortic and mitral valve calcifications Myocardial infiltrative damage	Vitamin D Calcium Avoid fruits and vegetables that affect cytochrome P450
Fabry	Renal failure or hematuria/proteinuria Angiokeratomas Acroparesthesias	Left ventricular hypertrophy Arrhythmias	Low fermentable oligosaccharide, disaccharide, monosaccharide and polyol (FODMAP) diet [62] Low-protein + keto analogs in patients with CKD
Pompe [23,63,64]	Muscle weakness Motor delay Feeding difficulties Recurrent respiratory infections	Hypertrophic cardiomyopathy Conduction abnormalities	Low-carbohydrate, high-protein diet [23] L-Alanine supplementation Aerobic exercise [65]
Niemann–Pick	Hepatosplenomegaly Ataxia Hypotonia Progressive severe neurologic impairment Difficulty with swallowing Severe liver disease	Cardiomegaly Endocardial fibroelastosis Valvular stenosis	Low-carbohydrate diet [66] Disaccharides restriction Ketogenic diet
MPS	Coarse features Skeletal abnormalities Neurologic impairment	Cardiac valve thickening Valvular dysfunction	Vitamins B1, B2, B3 Vitamin C Iron No specific diet indicated

**Table 4:** Summary of cardiac and systemic impact of different diets in patients with IMDs.

Diet Type	Cardiac Effect	Global Effects
Ketogenic	Decreases ventricular hypertrophy Improves ejection fraction	Seizure control Improves muscular function Reverses movement disorders Improves verbal response, memory, social interaction
FODMAP [62]	Potentially decreases cardiovascular disease risk (nonspecific impact on IMDs)	Ameliorates bowel function Decreases frequency of diarrhea, constipation, nausea
Low carbohydrate/high protein [3,64]	Inhibits cardiac remodeling caused by pressure overload (nonspecific impact on IMDs)	Improves muscle strength Increases exercise capacity

## Data Availability

No new data were created or analyzed in this study. Data sharing is not applicable to this article.
